# Addiction and the Dark Triad of Personality

**DOI:** 10.3389/fpsyt.2019.00662

**Published:** 2019-09-17

**Authors:** Emanuel Jauk, Raoul Dieterich

**Affiliations:** ^1^Clinical Psychology and Behavioral Neuroscience, Faculty of Psychology, Technische Universität Dresden, Dresden, Germany; ^2^Addiction Research, Faculty of Psychology, Technische Universität Dresden, Dresden, Germany

**Keywords:** Dark Triad, narcissism, Machiavellianism, psychopathy, substance use, substance use disorder, addiction

## Abstract

In this article, we review associations between the Dark Triad of personality (narcissism, Machiavellianism, and psychopathy) and addictive behaviors, both substance-related and non-substance-related. We summarize evidence from personality and clinical research and integrate it with prevailing models of addiction. Specifically, we discuss addictive behavior in the light of affect regulation, which is likely more relevant in narcissism, as well as inhibitory deficits, a putative mechanism in psychopathy. These mechanisms can be related to central motives of the respective personality constructs, such as stabilization of self-esteem in narcissism and impulsive stimulation seeking in psychopathy. We conclude that different mechanisms might lead to similar observable behavior in narcissism and psychopathy at earlier stages of the addiction cycle, but psychopathic disinhibition might be particularly relevant at later stages. This underpins the importance of considering personality factors for the understanding and treatment of addiction.

## The Dark Triad

The Dark Triad of personality—narcissism, Machiavellianism, and psychopathy (1)—attracted enormous research interest in the past decades. Given that the three traits are tied together by antagonism as a marker of emotionally cold interpersonal behavior (2), much of the pertinent literature focuses on intrinsically interpersonal topics such as workplace behavior, interpersonal attraction, or generally competitive contexts (3). The socially aversive, “dark” personality characteristics are partially related to short-term individual benefits in these contexts, such as vocational success [e.g., Ref. (4)] or mating success [e.g., Ref. (5)]. Besides these benefits, there are also significant costs. These encompass avoidant attachment [e.g., Ref. (6)]; dissatisfaction regarding needs for relatedness, competence, and autonomy; feelings of inauthenticity (7); reduced mental health^1^; risk-taking behavior; and—most important for the present review—substance use (8, 9).

We describe contemporary models of the three traits and review their associations with addictive behavior (substance-related and non-substance-related). We adopt a dimensional perspective, which assumes that the three traits display continuous distributions bending into clinically relevant personality configurations towards the upper ends. Additionally, we highlight findings from clinical groups with substance use disorders and/or personality disorders. The amount of available literature on the three traits differs substantially, with some literature for narcissism, little for Machiavellianism, and most for psychopathy. Among the three, it is mostly narcissism and psychopathy that display associations to substance use [e.g., Ref. (10), see **Table 1**]. Comorbidities of substance use disorders and the respective personality disorders are also well documented in community samples (11) and clinical groups (12, 13). The mechanisms promoting addictive behaviors in narcissism and psychopathy might differ and potentially target different phases of the addiction cycle: on the one hand, initially, instrumental use, driven by self-stabilizing motives, and on the other hand, compulsive use, characterized by loss of control despite negative consequences, which circumscribe fully developed substance use disorders (14).

**Table 1:** Summary of the reviewed literature on Dark Triad traits in relation to substance use and addictive behaviors.

	Narcissism	Machiavellianism	Psychopathy
Central characteristics	Self-importance and entitlement	Instrumental and manipulative behavior	Interpersonal–affective and antisocial–deviant traits
Primary broad trait	Antagonism	Antagonism	Antagonism
Secondary broad trait	Extraversion (grandiose)/neuroticism (vulnerable)	Unclear	Disinhibition (low conscientiousness)
Associations with substance use	+	∼	++
Motives for substance use	Regulation of self-esteem (grandiose and vulnerable), negative affect reduction (vulnerable)	Unclear	Stimulation seeking
Associations with substance use disorder	∼	Unclear	++
Associations with non-substance-related addictive behavior	++ (Robust evidence for problematic social media use)	Unclear	+ (Evidence for internet use and gambling)

“+” indicates evidence for positive association, “++” strong evidence for positive association, “∼” no evidence for association.

## Narcissism

Structural models of narcissism place self-importance and entitlement—both aspects of antagonism—at the core of the construct (15, 16). Beyond that, a grandiose form, a socially dominant, agentic–antagonistic interpersonal style, and a vulnerable form, a neurotic–antagonistic style, can be distinguished (16). In the general population, the grandiose and the vulnerable forms of narcissism are unrelated and display opposing characteristics with respect to psychological functioning and mental health, with grandiosity displaying (mostly) adaptive adjustment in self-report studies and vulnerability displaying signs of maladaptive adjustment and mental illness [cf. Ref. (17)]. Our recent studies suggest that both aspects might be intertwined at high grandiosity (18, 19). Concurrent grandiosity and vulnerability are referred to as pathological narcissism (20).

Grandiose narcissism is associated with substance use—particularly alcohol—in nonclinical groups (10, 21–25) and is elevated in substance use disorder groups (26). Vulnerable narcissism is also associated with substance use in nonclinical groups (21). Accordingly, pathological narcissism (concurrent grandiosity and vulnerability) was found to be substantially associated with alcohol and drug use (27, 28). Individuals with substance use disorders, compared to controls, display higher pathological narcissism, particularly in aspects of vulnerability such as entitlement rage,^2^ devaluing, hiding the self, and self-esteem contingency (30). Narcissistic personality disorder (defined as extreme grandiosity) is comorbid with alcohol and drug dependence (13). However, regarding dependence, narcissistic personality disorder does not necessarily have higher rates of comorbidity than other personality disorders (31, 32). Comorbidities might be explained by general functional impairment rather than specific characteristics of narcissism (33).

The putative mechanisms mediating substance use in narcissism are seen in self-regulatory functions, particularly the stabilization of self-esteem, which is high but instable in grandiose narcissism (34) and low in vulnerable narcissism (35). Drinking is related to grandiose narcissism and self-esteem contingency (such as need for approval) (23). Grandiose narcissism predicts drinking behavior independently of impulsivity, which indicates that other mechanisms might be relevant (24). This becomes even more apparent when grandiosity is accompanied by vulnerability, for which increased feelings of shame explain the association with problematic alcohol use (21).

Interestingly, in a recent study, an interpersonal aspect of pathological narcissism, *devaluing*, again turned out to be among the strongest predictors of impaired control over drinking and associated problems (36). Devaluing reflects “disinterest in others who do not provide needed admiration and shame over needing recognition from disappointing others” (37, p. 368). It could thus be speculated that this particular pattern of interpersonal avoidance goes hand in hand with substituting “real” others for “ideal” experiential states induced by the drug, such as long posited by psychoanalytic theorists: “Actually, the very term, ‘drug dependency’ reminds us of what we are dealing with, namely an archaic passive dependency on an all-giving, sempiternal, though narcissistically perceived—i.e., hugely inflated—object”^3^ (38, p. 838).

Beyond substance-related behavior, grandiose narcissism is linked to addictive social media use (24, 39–41), compulsive smartphone use (42), compulsive buying (43), pathological gambling (44), or compulsive working (45). Studies comparing grandiose narcissism to the other Dark Triad traits, however, do not always find effects for narcissism, but also point to the role of psychopathic or Machiavellian traits instead (46–49). The mechanisms that likely mediate the relationship between grandiose narcissism and problematic social media use are similar to those for substance use: grandiose narcissism is related to addictive Facebook usage *via* the need to be admired and—to a lesser extent—the need to belong (50). If their need for admiration is not satisfied, individuals with grandiose traits display more risk-taking behavior (51). In contrast to substance-related behavior (drinking), however, the associations between grandiose narcissism and disordered social media use can be explained by increased reward sensitivity (24), which points to the stronger involvement of approach-orientation.

Interestingly, in social anxiety disorder and avoidant personality disorder—which are highly comorbid with alcohol use disorder (52, 53)—interpersonal coping is also a frequently encountered motive for initial drinking. This highlights further parallels between social anxiety and vulnerable narcissism, which display substantial conceptual and empirical overlaps (54). Recent evidence suggests that socially anxious individuals who develop problematic or addictive drinking patterns may belong to a highly impulsive subtype of social anxiety disorder (55, 56). Future research on narcissism and addiction could integrate these findings to elucidate more fine-grained predictors of problematic or addictive substance use in narcissism.

Taken together, self-regulatory mechanisms—particularly the regulation of a chronically instable self-esteem—play a major role in the relation between narcissism and addictive behavior. For substance-related behaviors, this is most evident in substance use to cope with negative affect due to lack of others’ admiration and feelings of shame related to narcissistic vulnerability. For non-substance-related behaviors, using social media to feel admired might be a central mechanism.

## Machiavellianism

The concept of Machiavellianism was derived from Niccolò Machiavelli’s writings by the social psychologists Richard Christie and Florence Geis (57). In their conception, individuals displaying Machiavellian tendencies are characterized by instrumental and strategic interpersonal behavior alongside low orientation towards moral standards. In the Five Factor Model framework, Machiavellianism is—like narcissism and psychopathy—primarily characterized by interpersonal antagonism (58).

Christie and Geis (57) postulated that “Machs” should be characterized by the absence of psychopathology to allow for effective reality testing. This suggests that, among the three socially aversive traits, Machiavellianism should show no associations, or even negative associations, with addictive behavior. While empirical studies are scarce, Machiavellianism is indeed not significantly associated with global indices of substance use (10). Machiavellianism is, however, higher in cocaine users (59) and positively associated with indicators of problematic or addictive internet use (41, 47, 49), though not all studies find such associations (46, 48).

Beyond addictive behavior, empirical findings show that Machiavellianism is—depending on the scale and factor structure—not generally independent of psychopathology (60). However, some of these associations might be due to the multifaceted nature of the traditional Machiavellianism inventory, which also assesses low conscientiousness (58, 61). Recently, a new Machiavellianism scale was designed to assess the core characteristics in a purer fashion. Machiavellianism was uncorrelated with substance use and gambling; the “planful” aspect of Machiavellianism (positively associated with conscientiousness) was even negatively related to both (61), as could be expected on the basis of the original construct definition. To sum up, while more research will be needed for a comprehensive picture, existing studies do not point to a pivotal role of Machiavellianism in substance use, but suggest some associations with problematic internet use. Results may strongly depend on the operationalization of Machiavellianism, particularly the extent to which it draws on disinhibited (low-conscientious) aspects.

## Psychopathy

Structural models conceive psychopathy as a syndrome of interpersonal–affective and antisocial–deviant personality and behavior characteristics (62). These encompass, amongst others, an interpersonal style of superficial charm, grandiose self-worth (linking psychopathy to narcissism), manipulative behavior, shallow affect, and lack of empathy, as well as delinquency, stimulation seeking, and impulsivity. Regarding broad traits, psychopathy can be characterized mainly by interpersonal antagonism and aspects of low conscientiousness (disinhibition) (63–66). Traditional models of psychopathic traits in the general population also build upon the distinction between interpersonal–affective characteristics, also called factor 1 or primary psychopathic traits, and antisocial–deviant aspects, also referred to as factor 2 or secondary psychopathic traits (67). Alternative models propose two or three factors named fearless dominance/boldness, self-centered impulsivity/disinhibition, and coldheartedness/meanness (63, 68).

Psychopathic traits are reliably associated with substance use and addiction in forensic populations (12, 69–71) and also in the general population (10, 72, 73). The mechanisms that foster substance use and addiction in relation to psychopathic traits might differ from those of narcissism. Psychopathy—as outlined above—is associated with stimulation seeking and reduced inhibitory control with regard to potentially risky behavior [e.g., Ref. (10)]. Among the two factors, it is thus mainly the antisocial–deviant behavior, or secondary psychopathy, which is associated with substance use (12, 69, 71).

Neuroimaging work suggests that psychopathic—particularly antisocial–deviant—traits among healthy individuals are positively associated with striatal brain activity during monetary reward anticipation and application of amphetamine (74, 75), even when controlling for impulsivity. Given that similarly altered brain responses can predict problematic drug involvement (76), striatal hyperreactivity might facilitate drug use in highly risk-prone psychopathic individuals (but see evidence for striatal hyporeactivity as predictor of problematic drug use), (77). Drug users develop a sensitivity to substance cues, manifesting in increased activity in a circuit mediating reward, value, emotion, and salience processing, which is also related to subjective craving (78, 79). This is consistent with the incentive sensitization theory of addiction, which posits that pathologically high attribution of incentive salience to drug cues (“wanting/craving”), rather than the pleasurable effect of drugs (“liking”), drives compulsive drug use (80).

Interestingly, a neuroimaging study of the effects of drug cues in criminal offenders with a history of substance use disorders showed that characteristics of psychopathy negatively modulated brain responses to substance cues in this cue reactivity circuit (81). Modulation of brain responses was more pronounced for factor 2 (antisocial–deviant) than factor 1 (interpersonal–affective) psychopathy. A similar finding was recently obtained for adolescents: psychopathic characteristics negatively modulated neural cue reactivity, though in the youth sample, the negative association was more pronounced for factor 1 (82). However, most recently, a study on adult parolees with substance use disorder found evidence for a positive modulation of brain activation to drug cues by psychopathic traits (factor 1) (83). An important difference between this study and the prior investigations is the use of food cues rather than neutral stimuli as a control condition. The authors argue that individuals with higher psychopathic traits display stronger desensitization of non-drug-related cues. However, this effect was moderated by drug use history in such a way that highly psychopathic individuals with a longer drug use history showed lower sensitivity to drug cues (83).

Together, these findings suggest an interaction between psychopathic personality disposition and substance use: while those without a history of substance use display increased sensitivity to monetary and drug rewards, those with a longer history of substance use display decreased reactivity to drug cues. While it needs to be noted that these phenomena tap into different aspects of the addiction cycle, tentatively speaking, these studies suggest that cue reactivity or craving might not be the primary driving force of compulsive drug use in psychopathy. Other processes such as impulsivity or insensitivity to punishment, i.e., reduced behavioral control when assessing short-term benefits versus long-term risks or the implications of immediate negative feedback, might play a more prominent role. This would be consistent with recent longitudinal work (77) and addiction models conceptualizing compulsive drug use as the result of dysfunctional decision-making and learning processes (84, 85).

Psychopathic traits are also associated with non-substance-related addictive behaviors such as problematic social media or internet use (46–49) or problematic gambling (86–88) in the general population and in select populations, such as pathological gamblers [antisocial traits (89); for trait-level meta-analysis, see Ref. (90)]. In contrast to narcissism, there is little evidence for self-esteem stabilization or psychosocial coping as a functional mechanism.

Taken together, there is robust evidence for associations of substance use and addiction with psychopathic traits not only in forensic samples but also in the general population. Interestingly, these associations reflect a historic account to the classification of “anti-” as well as “dyssocial reactions” and alcohol/substance addictions, which were both subsumed under “sociopathic personality disturbance” in the first edition of the *Diagnostic and Statistical Manual of Mental Disorders* (*DSM*) [(91); see also Ref. (92)]. Unlike narcissism, there is little evidence for drug use as affect regulation in psychopathy. This aligns with the idea that individuals with psychopathic traits experience low levels of stress and anxiety, as for instance manifest in the negative correlations with neuroticism (64, 66). Substance use and addiction might be more related to stimulation seeking and impulsivity.

## Conclusion

As summarized in **Table 1**, narcissism and psychopathy are associated with substance-related and non-substance-related addictive behavior across nonclinical and clinical populations, whereas Machiavellianism is not. This aligns well with the view that narcissism and psychopathy can be placed on the *externalizing spectrum* of mental disorders alongside substance use disorders, as expressed in the Hierarchical Taxonomy of Psychopathology (HiTOP) (93). Beyond that, the HiTOP differentiates antagonistic-externalizing behavior, which characterizes narcissistic as well as antisocial traits, from disinhibited-externalizing behavior, which characterizes substance use disorders and antisocial traits. This model thus conceives antisocial traits in closer proximity to substance use than narcissistic traits, as they are tied together by disinhibited behavior (94). While this view is supported by clinical and nonclinical studies on psychopathic traits and addictive behavior, research on narcissism suggests links with substance use as well. This is in line with meta-analytic findings demonstrating that both disinhibition (linked to psychopathy) and antagonism (linked to narcissism and psychopathy) are related to substance-use disorders (95). The mechanisms promoting addictive behavior in association with narcissism and psychopathy might differ: individuals with narcissistic traits might be primarily driven by self-regulatory goals (i.e., affect regulation, stabilization of self-esteem), whereas disinhibition might foster substance use in relation to psychopathy. These mechanisms presumably target different phases of the addiction cycle. Self-regulatory goals might play a larger role in initial stages; impulsivity might be crucia to the development of fully developed substance use disorders.

## Author Contributions

Both authors contributed to the conceptualization and writing of this manuscript.

## Conflict of Interest Statement

The authors declare that the research was conducted in the absence of any commercial or financial relationships that could be construed as a potential conflict of interest.

## Footnotes

1 It needs to be noted, though, that the effects are heterogeneous for the three traits, and particularly narcissism, is also related to reports of subjective well-being.

2 Entitlement rage was considered a facet of grandiosity or vulnerability in different studies [see Ref. (29)].

3 The object, in psychoanalytic terms, refers to a real or imagined other, as a counterpart to the subject.
